# A retrospective register study comparing fibrinogen treated trauma patients with an injury severity score matched control group

**DOI:** 10.1186/s13049-019-0695-2

**Published:** 2020-01-21

**Authors:** Lou M. Almskog, Ulf Hammar, Agneta Wikman, Anders Östlund, Jonas Svensson, Michael Wanecek, Anna Ågren

**Affiliations:** 1 Department of Molecular Medicine and Surgery, Karolinska Institutet, Stockholm, Sweden; 2 Department of Anaesthesiology and Intensive Care, Capio St Görans Hospital, Stockholm, Sweden; 3 Unit of Biostatistics, Institute of Environmental Medicine, Karolinska Institutet, Stockholm, Sweden; 4 Department of Clinical Immunology and Transfusion Medicine, Karolinska University Hospital and Karolinska Institutet, Stockholm, Sweden; 5 Perioperative Medicine and Intensive Care, Karolinska University Hospital, Stockholm, Sweden; 6 Department of Physiology and Pharmacology, Section for Anesthesiology and Intensive Care Medicine, Karolinska Institutet, Stockholm, Sweden; 7 Centre for Psychiatry Research, Department of Clinical Neuroscience, Karolinska University Hospital and Karolinska Institutet, Stockholm, Sweden; 8 Department of Physiology and Pharmacology, Karolinska University Hospital and Karolinska Institutet, Stockholm, Sweden; 9 Department of Clinical Sciences, Division of Cardiovascular Medicine, Danderyd Hospital, Danderyd, 18288 Stockholm, Sweden; 10 Department of Medicine, Division of Hematology, Karolinska Institutet, Stockholm, Sweden

**Keywords:** Fibrinogen, Trauma, Transfusion, Coagulopathy

## Abstract

**Background:**

Fibrinogen concentrate (FC) is frequently used to treat bleeding trauma patients, although the clinical effects are not well known. In this study we describe demographic and clinical outcome data in a cohort of trauma patients receiving FC, compared to a matched control group, who did not receive FC.

**Methods:**

This retrospective, single-center, observational study included adult trauma patients admitted to a level 1-trauma center in Sweden between January 2013 and June 2015. The study population consisted of patients to whom FC was administrated within 24 h (*n* = 138, “Fib+”). Patients with Injury Severity Score (ISS) > 49 and/or deceased within 1 h from arrival were excluded (*n* = 30). Controls (*n* = 108) were matched for age, gender and ISS (“Fib-“). Primary outcome was mortality (24 h−/30 days−/1 year-), and secondary outcomes were blood transfusions, thromboembolic events and organ failure.

**Results:**

The Fib+ group, despite having similar ISS as Fib-, had higher prevalence of penetrating trauma and lower Glasgow Coma Scale (GCS), indicating more severe injuries. Patients receiving FC had a higher mortality after 24 h/ 30 days/ 1 year compared to controls (Fib-). However, in a propensity score matched model, the differences in mortality between Fib+ and Fib- were no longer significant. Blood transfusions were more common in the Fib+ group, but no difference was observed in thromboembolic events or organ failure. In both groups, low as well as high P-fibrinogen levels at arrival were associated with increased mortality, with the lowest mortality observed at P-fibrinogen values of 2–3 g/l.

**Conclusions:**

Despite equal ISS, patients receiving FC had a higher mortality compared to the control group, presumably associated to the fact that these patients were bleeding and physiologically deranged on arrival. When applying a propensity score matching approach, the difference in mortality between the groups was no longer significant. No differences were observed between the groups regarding thromboembolic events or organ failure, despite higher transfusion volumes in patients receiving FC.

## Background

The fibrinogen protein plays a crucial role in blood coagulation and fibrinolysis. During blood loss, fibrinogen has been reported to decrease more rapidly towards critically low concentrations compared to other coagulation factors [1, 2]. In the healthy individual the range of plasma fibrinogen is 2–4 g/l, and at levels below this range the fibrinogen concentration may be too low to adequately support the construction of a functional haemostatic clot [3, 4]. Currently, Fibrinogen concentrate (FC) is approved for treatment and prophylaxis of acquired and congenital fibrinogen deficiency [5], and European guidelines for massive haemorrhage following trauma recommend substituting fibrinogen at concentrations below 1.5 to 2.0 g/l. [6]

Trauma-induced coagulopathy (TIC) is an early-onset syndrome characterized by an endogenously impairment of haemostasis, associated with a 4-fold higher mortality, increased transfusion requirements and organ failure [7, 8]. TIC is primarily characterized by a reduction in clot strength, and studies have highlighted the pathogenic contributions of shock and tissue injury resulting in systemic anticoagulation, hyperfibrinolysis and fibrinogen depletion. However, controversy exists over the precise mechanism of this syndrome, and failure to define the pathophysiology of TIC has prevented identification of the optimal therapeutic intervention [9, 10]. Within the last decade, research focusing on TIC has led to improved resuscitation strategies, mainly through early and more aggressive use of blood products and coagulation factors [11].

Several recent studies have indicated that fibrinogen and other coagulation factors are rapidly consumed after trauma, and that this phenomenon has a strong association to increased mortality [7, 12]. Decreased plasma fibrinogen concentration shortly after injury is associated with higher blood transfusion needs and increased mortality, and patients with coagulation abnormalities are known to develop organ dysfunction and spend longer time in the intensive care unit [13, 14]. Early supplementation with fibrinogen concentrate in severe traumatic haemorrhage is a theoretically appealing therapeutic option, but has not yet been shown to significantly improve clinical outcome in randomized studies [13, 15]. Though there are randomized trials running [16, 17] the current evidence on the use of FC in trauma is mostly restricted to retrospective analyses. Despite the fact that the beneficial effect of treatment with FC is still debated, many trauma centers around the world have implemented the administration of FC to bleeding trauma patients in their transfusion protocols [13, 18].

Elevated levels of P-fibrinogen are associated with increased risks in general, and may even amplify the effects of other established cardiovascular risk factors, contributing to a patient’s acute risk of cardiovascular disease [19]. Therefore, high plasma levels of P-fibrinogen after trauma could be hypothesized to function as a predictor for poor clinical outcome.

The aim of this study was to 1) describe the cohort of trauma patients receiving FC at the Trauma Center of Karolinska University Hospital (TCK); 2) compare demographic and outcome data for patients given FC to a control group matched for age, sex and Injury Severity Score (ISS); 3) evaluate whether low P-fibrinogen on arrival could be confirmed to predict mortality.

## Methods

### Study design

This descriptive, retrospective register study of adult trauma patients treated at the TCK, the Stockholm region’s level 1 trauma unit and Sweden’s largest trauma center, was conducted between January 2013 and June 2015. TCK is covering an area with approximately 2.5 million people. More than 1800 trauma cases are treated per year. The majority of patients (80%) are not severely injured (ISS < 15).

**Fig. 1:**
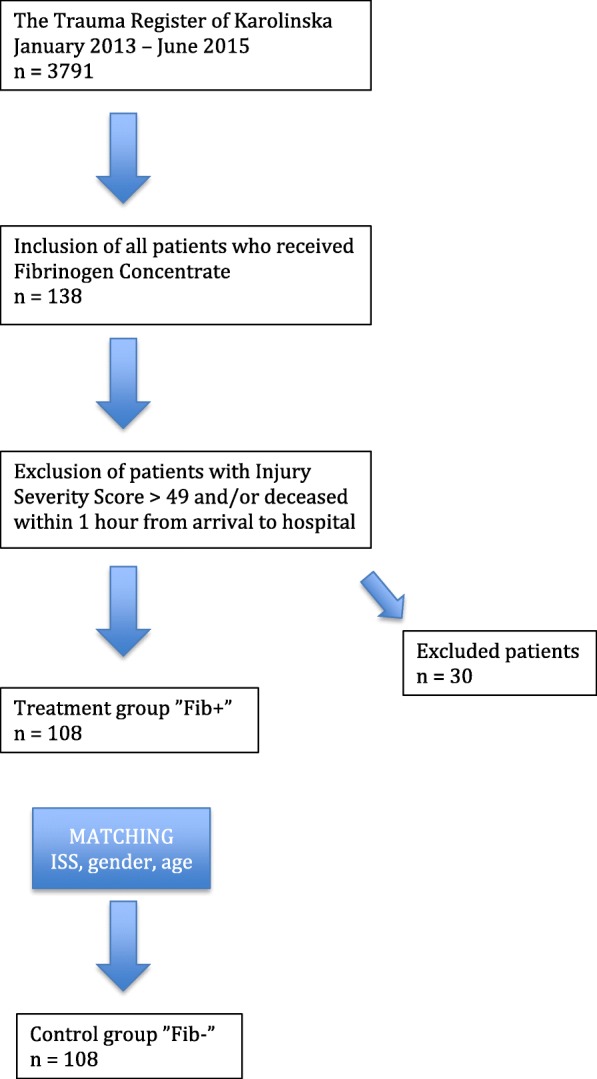
Study design. Inclusion/exclusion criteria and matching criteria of the control group. Patients receiving FC (Fib+), patients not receiving FC (Fib-)

Patients receiving fibrinogen concentrate (FC) (RiaSTAP™, CSL Behring, Germany) were included and compared to a control group, that did not receive FC. The control group was matched for age, sex and ISS and was treated at the same hospital during the same time period. See Fig. 1. The primary outcome of the study was mortality, and the secondary outcomes were transfusion volumes, thromboembolic events and organ failure.

### Study population

#### Fibrinogen group (Fib+)

During a period of 2.5 years (January 1st 2013 to June 30th 2015) 3791 patients admitted to TCK were recorded in The Trauma Register of Karolinska (TRK). Patients > 18 years to whom FC was administrated within 24 h from arrival to hospital, were included in the study (*n* = 138). Patients with Injury Severity Score (ISS) > 49 and/or deceased within 1 h were excluded (*n* = 30) to avoid survival bias. All excluded patients also received treatment with FC (see Additional file 1: Table S5).

#### Control group (Fib-)

A control group (*n* = 108) with matching criteria of age, sex and ISS was obtained from TRK. An algorithm written in the programming language R (version 3.3.3) was used to identify all matching patients and then randomly draw one control for each subject. All control patients were treated at TCK during the same time period as Fib+. The electronic medical records were retrospectively reviewed for each patient in both groups, and demographic and clinical data prior to/during the initial resuscitation were collected. All blood samples were drawn on arrival to hospital, before administration of any transfusions or factor concentrates. For a complete list of collected variables, see Table 1.

**Table 1:**
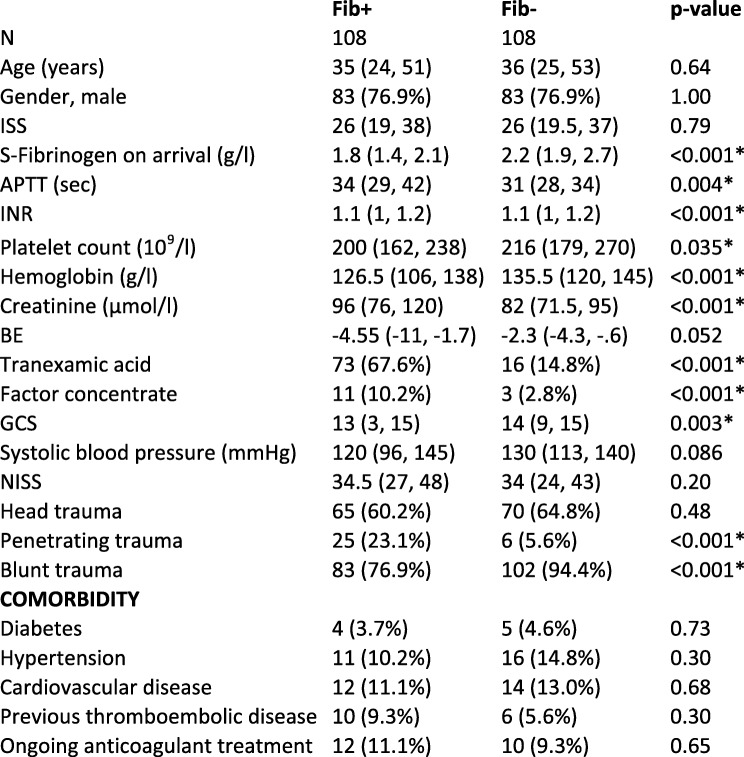
Demographics for treatment group and controls on admission

Median (IQR), Number (%), * Significant values. Matching criteria age-gender-ISS at the top. Injury Severity Score (ISS), Activated Partial Thromboplastin Time (APTT), International Normalized Ratio (INR), Platelet count, Base Excess (BE), Glasgow Coma Scale (GCS), New Injury Severity Score (NISS). Continuous variables were analysed with the Mann-Whitney test and categorical variables with Fisher’s exact test

### The trauma register of Karolinska

The Trauma Register of Karolinska (TRK) is a local register of trauma patients treated at TCK, where inclusion criteria is assumed traumatic injury of any kind and/or ISS > 9. It covers demography, patient factors, prehospital details, outcome and quality of hospital care. Exclusion criteria are isolated fractures of the upper/lower limb. Additionally, information concerning given blood products and/or coagulation factors (fibrinogen concentrate, prothrombin complex concentrate, factor VIII/von Willebrand factor, factor VII) is entered in the register.

### Logistics for treatment of massive bleeding at the trauma Center of Karolinska

At the Trauma Center of Karolinska University Hospital (TCK), FC is administrated to selected trauma patients, based on the decision of the attending anaesthesiologist, who also is responsible for initiating the massive bleeding protocol if appropriate.

The standardized massive bleeding protocol at Karolinska University Hospital consists of RBC:Plasma:Platelets, ratio 4:4:1 units (one platelet unit is either from a pool of four buffy-coats or one apheresis unit), Fibrinogen concentrate, 2–4 g (30–40 mg/kg), Tranexamic acid, 2 g and Calcium if ionized Ca < 1,0 mmol/l.

A standardized clinical approach consisting of a primary survey according to the Advanced Trauma Life Support (ATLS) concept is performed, and a standard pack of blood samples including arterial blood gas, blood count, PK/INR, APTT and P-fibrinogen is collected.

### The transfusion register of the transfusion medicine unit

The information of blood transfusions was retrieved from the blood data system ProSang (CSAM Software Solutions, Lysaker, Norway), at the department of Transfusion Medicine, Karolinska University Laboratory.

### Trauma induced coagulopathy

A subanalysis including patients with hypocoagulability on admission was performed, where hypocoagulability was defined as an International Normalized Ratio (INR) > 1.1 (Triolab AB®, Stockholm, Sweden, reference value < 1.2) and/or Activated Partial Thromboplastin Time (APTT) > 40 s (Triolab AB®, Stockholm, Sweden, reference value 28–40 s) and/or P-fibrinogen < 2 g/l (Dade Behring/Siemens®, Munich, Germany, reference value 2–4.2 g/l) and were analyzed with the Sysmex CS 2100i® and Sysmex XE 5000/XT 2000i® system (Kobe, Japan) in the Department of Clinical Chemistry, Karolinska University Hospital Solna.

As there is no clear consensus regarding an approach to the classification of trauma-associated coagulation impairment [20], our definition of hypocoagulability was simply based on coagulation laboratory values out of reference range, as stated above.

### Ethical approvement

The study was approved by the regional ethical review board in Stockholm, Sweden (Dnr 2007/371–31/3).

### Statistical analysis

Baseline differences between Fib+ and Fib- were analysed with the Mann-Whitney test (for continuous variables) or Fisher’s exact test (for discrete variables). The average treatment effect on the treated (ATET) of FC supplementation was analysed using propensity score matching. Outcomes were 24 h-, 30 days- and 1 year-mortality and RBC-, platelet-, plasma- and total transfusions. The propensity score model included age, sex, ISS, baseline value of P-fibrinogen, Activated Partial Thromboplastin Time (APTT), International Normalized Ratio (INR), Platelet count, hemoglobin, creatinine, Glasgow Coma Scale (GCS) and penetrating trauma. An additional model also included diabetes, hypertension, cardiovascular disease, thromboembolism and chronic obstructive pulmonary disease in the propensity score model. A subanalysis was performed for patients with hypocoagulability on arrival. All our *p*-values come from two-tailed tests, and our alfa-level was pre-set to 0.05. No correction for multiple comparisons (Bonferroni correction) was made.

As a secondary analysis, the ability of each covariate to predict mortality was investigated. This analysis was performed using univariate logistic regressions, with 24 h-, 30 days- and 1 year-mortality as outcomes. Predictive ability was evaluated using the area under the curve (AUC), as calculated from 10-fold cross-validation. In this analysis, all continuous covariates were modelled using restricted cubic splines. Creatinine was analysed as the difference from the reference interval, to account for the fact that reference values of creatinine differ depending on gender. A similar analysis was performed to predict transfusion levels (plasma, platelet, RBC and total). Here predictive ability was evaluated using R^2^-values.

## Results

### Demographics and matching criteria

23.1% of included patients were female. The median age was 35 [IQR 24; 51] (Fib+) vs. 36 [IQR 25; 53] (Fib-). 76.9% were exposed to blunt trauma, 23.1% to penetrating trauma and 60.2% to head trauma in the Fib+ group. For further demographic data, laboratory test results and comorbidities, see Table 1. For demographic data of excluded patients (*n* = 30), see Additional file 1: Table S5.

### P-fibrinogen and other laboratory tests on arrival

P-fibrinogen on arrival was lower in Fib+ compared to controls (1.8 g/l (median) [IQR 1.4; 2.1] vs. 2.2 g/l [1.9; 2.7], (*p* < 0.001). A median of 2 g of fibrinogen concentrate (FC) (RiaSTAP™, CSL Behring, Germany) [IQR 2; 3] was given within the first 24 h after admission to the Fib+ group. Higher Activated Partial Thromboplastin Time (APTT), higher International Normalized Ratio (INR), lower platelet count, lower haemoglobin and higher creatinine were observed in Fib+. INR was significantly different in Fib+ compared to Fib- (see Table 1), although the median and IQR were similar. More patients in Fib+ received tranexamic acid and factor concentrates (Prothrombin complex concentrate and/or recombinant FVII (NovoSeven®; Novo Nordisk, Denmark) and/or FVIII+Von Willebrand factor (Haemate®, CSL Behring, Germany). For complete details, see Table 1.

### ISS in study population

Median ISS was 26 [IQR 19; 38] in Fib+ and 26 [IQR 19.5; 37] in Fib-, (*p* = 0.79). Penetrating trauma was more common in the Fib+ group, 23.1% compared to 5.6% in Fib- (*p* < 0.001). 60.2% in Fib+ were exposed to head trauma, vs. 64.8% in Fib- (*p* = 0.48). Patients in Fib+ had lower Glasgow Coma Scale (GCS) on arrival (12.5 [IQR 3; 15]) compared to controls (14 [IQR 9; 15], *p* = 0.003).

### Outcomes

#### Mortality

**Fig. 2:**
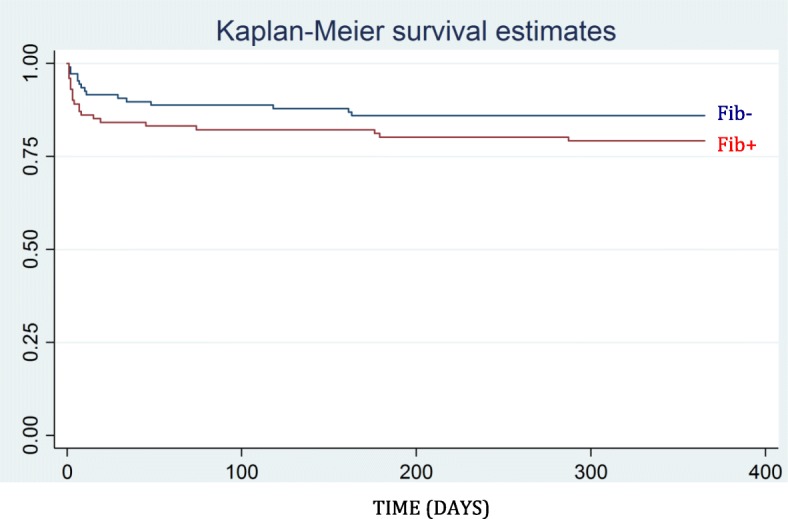
Kaplan-Meier survival estimates, 1 year. Survival among patients receiving FC (Fib+) compared to controls (Fib-)

Patients receiving FC had a higher mortality after 24 h/ 30 days/ 1 year compared to controls (Fib-): 24 h-mortality was 6.5% vs 0.9% (95% CI 0.6–10.5, *p* = 0.029), 30 days-mortality 21.3% vs 10.2% (95% CI 1.5–20.7, *p* = 0.023), 1 year-mortality 25.9% vs 14.8% (95% CI 0.5–21.8, *p* = 0.041), where all confidence intervals represent the differences between the groups. Survival is further presented in Fig. 2. Patients in Fib+ were more physiologically deranged, had higher percentage of penetrating injuries and needed more blood transfusions. In an attempt to adjust for this imbalance, a propensity score matching approach was applied. In this analysis the difference in mortality was no longer significant (see Additional file 1: Table S7).

#### Transfusions

More RBC (5 units [2; 9]^1^ vs. 0 units [0; 2]^1^, *p* < 0.001), plasma (6 units [2; 10]^1^ vs. 0 units [0; 2]^1^, *p* < 0.001) and platelets (1 unit [0; 2]^1^ vs. 0 units [0; 0]^1^, *p* < 0.001) were administrated to Fib+ compared to Fib- within the first 24 h.

No significant differences were observed regarding arterial/venous thrombosis, Acute Respiratory Distress Syndrome (ARDS), Multiple Organ Failure (MOF), Transfusion-Related Acute Lung Injury (TRALI) or Acute Kidney Injury (AKI), see Table 2.

**Table 2:**
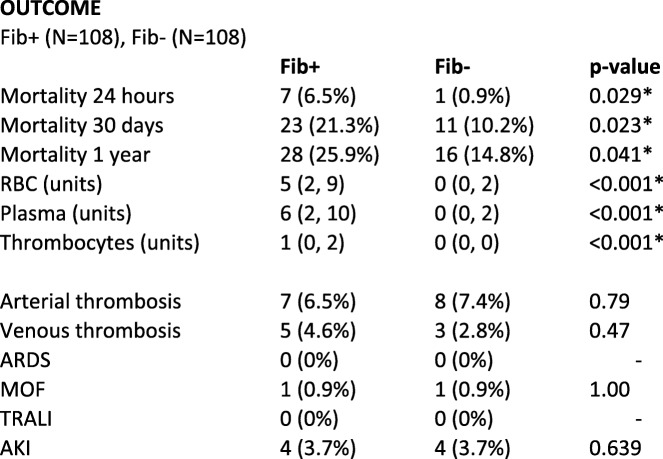
Outcome, treatment group and controls

Median (IQR), Number (%), * Significant values. Transfusions given within the first 24 h from arrival to hospital. Red Blood Cells (RBC), Acute Respiratory Distress Syndrome (ARDS), Multiple Organ Failure (MOF), Transfusion-Related Acute Lung Injury (TRALI), Acute Kidney Injury (AKI). Continuous variables were analysed with the Mann-Whitney test and categorical variables with Fisher’s exact test

#### Predicting factors for mortality

High creatinine turned out to be the best predictor for 24 h-mortality (Area Under the Curve = AUC 0.79 [95% CI 0.63; 0.94]), low GCS the best predictor for 30 days-mortality (AUC 0.73 [95% CI 0.63; 0.83]) and high APTT the best predictor for 1 year-mortality (AUC 0.71 [95% CI 0.62; 0.79]). Hence, these were all better predictors than other variables such as ISS, penetrating trauma, INR and hemoglobin. For AUC-values of different predictors of mortality, see Additional file 1: Table S11.

#### Fibrinogen level on arrival predicting mortality

**Fig. 3:**
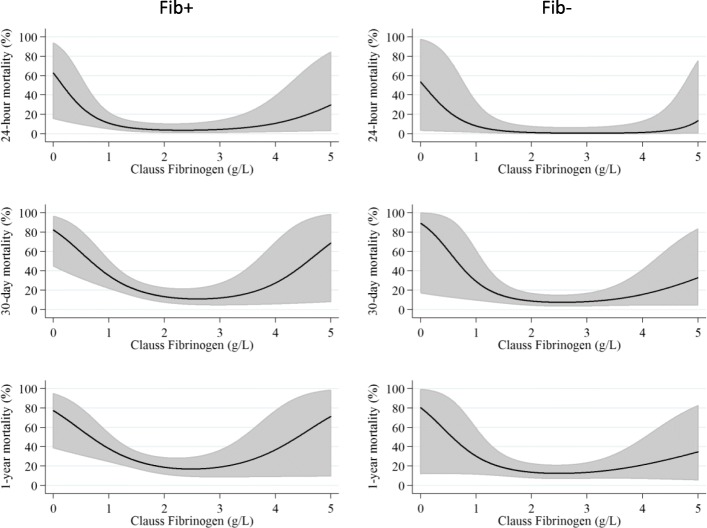
Mortality (24 h−/30 days−/1 year-) vs. P-fibrinogen (g/l) on arrival to hospital for patients receiving FC (Fib+, left row) and controls (Fib-, right row)

Mortality vs. P-fibrinogen on arrival is illustrated in Fig. 3. In both Fib+ and Fib-, low P-fibrinogen levels were associated with increased mortality, as well as high P-fibrinogen levels, in a biphasic manner. Lowest mortality was seen at P-fibrinogen values of 2–3 g/l. When adjusting for confounders (age, ISS, APTT, INR, hemoglobin, creatinine, GCS) the biphasic shape of the curve remained (see Additional file 1: Figure S4).

Furthermore, patients were divided into subgroups based on P-fibrinogen levels on arrival (cases/controls), where low P-fibrinogen was defined as < 2 g/l, normal levels as 2–3 g/l and high P-fibrinogen as > 3 g/l. Patients with low P-fibrinogen were younger (34.4 years [IQR 23.3; 49.9]), had low GCS (median 7 [IQR 3; 14]) and 11% had pre-existing cardiovascular disease. Patients with high P-fibrinogen were older (69.5 years [IQR 57.0; 80.5]), had high GCS (15 [IQR 15; 15]) and 75% had pre-existing cardiovascular disease. See Additional file 1: Tables S8 and S9.

#### Predicting factors for transfusion

Hemoglobin was the best predictor for all transfusions; RBC transfusion (coefficient of determination (R^2^) =0.11), plasma transfusion (R^2^ = 0.11), platelet transfusion (R^2^ = 0.12) and total transfusions (RBC + plasma + platelets, R^2^ = 0.12). R^2^-values for all predictors of transfusion are presented in Additional file 1: Table S12.

### Trauma induced coagulopathy

One hundred forty-eight patients (90 patients in Fib+ and 58 controls) met the criteria of hypocoagulability on arrival defined for this study (INR > 1.1 and/or APTT > 40 s and/or P-fibrinogen < 2 g/l). Patient characteristics of this subanalysis (patients with coagulopathy) showed essentially the same patterns as the main analysis (all patients) in terms of age, sex and ISS. The distribution of trauma mechanisms (head/penetrating/blunt trauma) in the subanalysis vs. the main analysis was also comparable, as were the blood transfusion volumes. Mortality was high in the hypocoagulable cohort. For demographic data of this subanalysis, see Table 3.

**Table 3:**
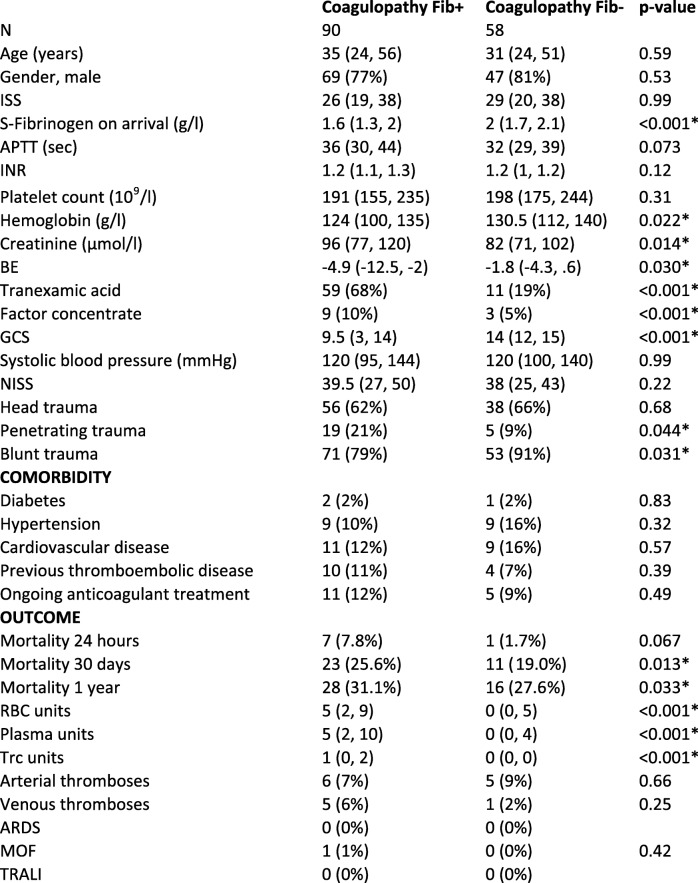
Demographics and outcome, coagulopathic patients, treatment group and controls

Patients fulfilling the criteria of coagulopathy on arrival. Criteria of coagulopathy defined for this study: INR > 1.1 and/or APTT > 40 s and/or P-fibrinogen < 2 g/l. Median (IQR), Number (%), * Significant values. Transfusions given within the first 24 h from arrival to hospital. Matching criteria age-gender-ISS at the top. Injury Severity Score (ISS), Activated Partial Thromboplastin Time (APTT), International Normalized Ratio (INR), Platelet count, Base Excess (BE), Glasgow Coma Scale (GCS), New Injury Severity Score (NISS), Red Blood Cells (RBC), Thrombocytes (Trc), Acute Respiratory Distress Syndrome (ARDS), Multiple Organ Failure (MOF), Transfusion-Related Acute Lung Injury (TRALI). Continuous variables were analysed with the Mann-Whitney test and categorical variables with Fisher’s exact test

### ROTEM analysis

Rotational Thromboelastometry (ROTEM) data was obtained for 33 patients in Fib+ and 32 controls. A significant difference in FIBTEM-MCF on arrival was observed between Fib+ and Fib- 12 [9; 15] vs. 13.5 [11; 18] (*p* = 0.045). For the remaining ROTEM parameters, there was no significant difference observed. ROTEM variables are listed in Additional file 1: Table S6.

## Discussion

In this study we have examined the mortality and transfusion volumes among trauma patients receiving FC in comparison to matched controls in a retrospective cohort.

We observed that trauma patients to whom FC was administrated within 24 h from arrival (Fib+) had a higher mortality and received more transfusions compared to controls. Although Injury Severity Score (ISS) was comparable, the Fib+ group presented signs of being more severely injured already on arrival. Conceivably, the increased mortality and transfusion requirements in Fib+ do not seem to be because of the FC treatment, but rather due to a higher incidence of haemorrhagic shock among these patients. After propensity score matching the difference in mortality was no longer significant.

We observed no difference in the incidence of Acute Respiratory Distress Syndrome (ARDS), Multiple Organ Failure (MOF) or Acute Kidney Injury (AKI) between the Fib+ and Fib- groups (Table 2). This is surprising, since an association between high transfusion volumes and ARDS/MOF has been shown [21].

The lack of difference between the groups regarding ARDS/MOF incidence could be confounded by survival bias, as organ failure won’t affect non-survivors in the same extent as survivors.

Our dataset may illustrate a situation where patients, although presenting with the same ISS, suffer from different injury panoramas with varying degrees of physiologic instability, not captured by the ISS system, such as bleeding and hypocoagulability. In an attempt to adjust for this potential selection bias we used a propensity score weighted model, where we adjusted for factors that hypothetically could capture the imbalance between the groups (age, sex, ISS, baseline value of P-fibrinogen, APTT, INR, platelet count, hemoglobin, creatinine, GCS and penetrating trauma). In this analysis, the Fib+ and Fib- groups were similar with regards to mortality outcomes.

Low fibrinogen concentration (< 1.5 g/l) on admission has been associated with an increased risk of death in trauma patients requiring massive transfusion [22]. In our data, we additionally observed that *high* P-fibrinogen on arrival correlated with increased mortality. A possible explanation for high fibrinogen levels in trauma patients, could be that fibrinogen is considered as an acute phase reactant [23]. High P-fibrinogen levels have been shown to increase the risk of cardiovascular events, especially in patients already suffering from cardiovascular disease [19].

Since FC typically is administrated before lab results are accessible, some patients receive FC in spite of high P-fibrinogen levels. How patients with already high levels of P-fibrinogen on arrival respond to administration of FC remains unclear. But, as fibrinogen activates key players of the inflammatory response [24], high levels of P-fibrinogen at arrival may thus reflect a patient with a high inflammatory profile, who may not benefit from administration of FC. The association between high levels of P-fibrinogen and increased mortality may underscore the importance of individualized haemostatic therapy after trauma.

In an attempt to classify whether specific P-fibrinogen levels were associated with certain patient characteristics, subgroup analyses were performed where patients were divided into subgroups based on P-fibrinogen levels on arrival (cases/controls). In the Fib+ group, low P-fibrinogen levels (< 2 g/l) were associated with young age and only a few had prior cardiovascular comorbidity. Furthermore, several in this group suffered from major haemorrhage. High P-fibrinogen levels (> 3 g/l) on the other hand, were associated with high age and cardiovascular comorbidities. This is in line with previously published results, showing that the baseline fibrinogen level is higher in older patients, increasing by approximately 0.01 g/L per year of age [25]. This could be interpreted as reflecting the fact that high age and co-existing disease in this group makes it less likely to survive a trauma. However, the persisting biphasic shape of the curve in the adjusted material (Additional file 1: Figure S4) indicates that the association is not that simple.

### Predictors

Of the possible predictors for clinical outcome we examined in our data, creatinine was the best predictor of 24-h mortality. This may represent sensibility for renal hypoperfusion caused by haemorrhagic shock. AKI is an uncommon but serious complication after trauma [26]. In early literature, AKI is mainly reported secondary to crush injuries and rhabdomyolysis, associated with a significant risk of morbidity and mortality [27]. It is well known that damage of skeletal muscle can cause release of myoglobin, initiating the pathophysiologic process associated with rhabdomyolysis [28]. An early peak in S-creatinine following trauma may be due to muscular injury or decreased perfusion pressure causing an increase in muscle breakdown [29]. Few patients in our study met the criteria for AKI.

Glasgow Coma Scale (GCS) was the best predictor for 30-days mortality. Multiple studies have reported the predictive value of the GCS, alone or in combination with other clinical factors, in determining the outcome of patients who have sustained brain injury. Kung et al. showed the GCS to be predictive for the survival of traumatic brain injury (TBI) patients [30].

Activated Partial Thromboplastin Time (APTT) was the best predictor for 1-year mortality. MacLeod et al. concluded that coagulopathy as defined by an elevated APTT plays a major role in early trauma-related deaths [31].

### Bias

Survival bias is a potential problem in our study. One may consider, whether a probable delay of administration of FC might have excluded the most severely injured patients from the Fib+ group. This circumstance would exaggerate the benefit of FC treatment, as only “survivors” would get treatment. Reversely, the “best” patients might have recovered before FC was given, and thus gotten excluded from Fib+, contributing to better outcomes in Fib- [32].

Stratification of patients according to ISS has been applied previously in the literature [33], and to reduce the risk of survival bias in our study, the most severely injured patients with multiple trauma (ISS > 49 and/or deceased within 1 h from arrival) were excluded. In our data, few controls had ISS > 49, especially in the older age categories, making the matching process challenging. Exclusion criteria were based on studies demonstrating that patients with ISS > 49 have the poorest odds of survival, as over 70% die within the first hour after arrival to hospital, and 90% within 6 h [34]. However, in our material the mortality was lower, which might be due to a different study population.

### Limitations and strengths

This study has limitations: the sample size is relatively small with few events. The majority of deaths are observed among hypocoagulable patients in the Fib+ group, however, the effects of individual components of the resuscitation strategy are difficult to interpret, as a huge number of contributing factors could have an impact on mortality in our patient cohorts.

As the difference in mortality between our two groups no longer was significant after adjusting for important factors (e.g. baseline value of S-fibrinogen, APTT, INR, Platelet count, hemoglobin, creatinine, GCS, penetrating trauma), it is possible that the association between FC and mortality would have been made more clear by including additional coagulopathy-related aspects in the propensity score model.

One strength of the study is the thorough extraction of data from the patient medical records, completing the register data.

It has been suggested that the ISS system may be underestimating the volume of tissue injury sustained in patients with multiple injuries [14]. In line with this, our results indicate that the severity of injury was overestimated in the control group, as these patients were less physiologically deranged on arrival to hospital compared to the Fib+ group, in spite of similar ISS. This may implicate the need of an injury classification system where hypocoagulability and bleeding are taken into account. In future studies, validation of other scoring systems meeting these requirements would be of great interest.

## Conclusions

Patients receiving fibrinogen concentrate had a higher mortality compared to a matched control group with equal ISS, presumably caused by the fact that these patients were bleeding and physiologically more deranged on arrival. When applying a propensity score matching approach, the difference in mortality between the groups was no longer significant. Creatinine, GCS and APTT did more accurately predict mortality compared to ISS. Further, in our data, both low and high P-fibrinogen on admission was associated with increased mortality.

## Supplementary information

**Additional file 1: Table S5.** Demographics and outcome, excluded patients. All these patients received treatment with FC. **Table S6.** ROTEM data. **Table S7.** Average treatment effect on the treated (ATET), crude and adjusted. **Table S8.** S-fibrinogen intervals, treatment group. **Table S9.** S-fibrinogen, intervals, controls. **Table S10.** Outcome, subanalysis coagulopathy. **Table S11.** AUC-values for different predictors of mortality (univariate analysis). **Table S12.** R2-values for different predictors of transfusion (total units of RBC/plasma/thrombocytes/total transfusions). **Table S13.** AUC-values for different predictors of mortality (univariate analysis), coagulopathic patients. **Figure S4.** 30 days-mortality vs. S-fibrinogen (g/l), all patients. Data adjusted for confounders (age, Injury Severity Score (ISS), Activated Partial Thromboplastin Time (APTT), International Normalized Ratio (INR), hemoglobin, creatinine, Glasgow Coma Scale (GCS).

## Data Availability

The datasets analysed during the current study are available from the corresponding author on reasonable request.

## Abbreviations

AKI: Acute Kidney Injury
APTT: Activated Partial Thromboplastin Time
ARDS: Acute Respiratory Distress Syndrome
ATLS: Advanced Trauma Life Support
ATT: The Average Treatment effect for the Treated
AUC: The Area Under the Curve
BC: The Transfusion Medicine Unit, Blodcentralen Karolinska
CT: Computed Tomography
FC: Fibrinogen concentrate
FIBTEM-MCF: Maximum Clot Firmness of a Fibrinogen Thromboelastometry
GCS: Glasgow Coma Scale
GOS: Glasgow Outcome Scale
INR: International Normalized Ratio
ISS: Injury Severity Score
MOF: Multiple Organ Failure
PC: Platelet Count
R2: Coefficient of determination
RBC: Red Blood Cells
ROTEM: Rotational Thromboelastometry
SweTrau: The Swedish national Trauma register
TCK: The Trauma Center of Karolinska University Hospital
TEG: Thromboelastography
TIC: Trauma-induced coagulopathy
TR: The Trauma Register of Karolinska
TRALI: Transfusion-Related Acute Lung Injury

## Footnotes

1 Median [IQR]
