# Supplementary material for: A retrospective register study comparing fibrinogen treated trauma patients with an injury severity score matched control group
Source: Scand J Trauma Resusc Emerg Med. 2020 Jan 21;28:5. doi: 10.1186/s13049-019-0695-2 (PMC6975055; doi:10.1186/s13049-019-0695-2)
Supplement: Supplementary file 1 — Additional file 1: Table S5. Demographics and outcome, excluded patients. All these patients received treatment with FC. Table S6. ROTEM data. Table S7. Average treatment effect on the treated (ATET), crude and adjusted. Table S8. S-fibrinogen intervals, treatment group. Table S9. S-fibrinogen, intervals, controls. Table S10. Outcome, subanalysis coagulopathy. Table S11. AUC-values for different predictors of mortality (univariate analysis). Table S12. R2-values for different predictors of transfusion (total units of RBC/plasma/thrombocytes/total transfusions). Table S13. AUC-values for different predictors of mortality (univariate analysis), coagulopathic patients. Figure S4. 30 days-mortality vs. S-fibrinogen (g/l), all patients. Data adjusted for confounders (age, Injury Severity Score (ISS), Activated Partial Thromboplastin Time (APTT), International Normalized Ratio (INR), hemoglobin, creatinine, Glasgow Coma Scale (GCS). [file 13049_2019_695_MOESM1_ESM.docx]

Additional file 1

Table 5:

*Demographics and outcome, excluded patients. All these patients received treatment with FC.*

**

*Median (IQR), Number (%). Transfusions given within the first 24 hours from arrival to hospital. Matching criteria age-gender-ISS at the top. Injury Severity Score (ISS), Activated Partial Thromboplastin Time (APTT), International Normalized Ratio (INR), Platelet count, Base Excess (BE), Glasgow Coma Scale (GCS), New Injury Severity Score (NISS), Chronic Obstructive Pulmonary Disease (COPD), American Society of Anesthesiologists Physical Status Classification System (ASA), Red Blood Cells (RBC), Acute Respiratory Distress Syndrome (ARDS), Multiple Organ Failure (MOF), Transfusion-Related Acute Lung Injury (TRALI).*

Table 6:

*ROTEM data.*

**

*Median (IQR), * Significant result. All variables were analysed with the Mann-Whitney test.*

Table 7:

*Average treatment effect on the treated (ATET), crude and adjusted.*

**

** Significant results. Adjusted confounders: age, sex, Injury Severity Score (ISS), S-fibrinogen, Activated Partial Thromboplastin Time (APTT), International Normalized Ratio (INR), Platelet count, hemoglobin, creatinine, Glasgow Coma Scale (GCS) and penetrating trauma. Adjusted comorbidities: diabetes, hypertension, cardiovascular disease, thromboembolism and Chronic Obstructive Pulmonary Disease (COPD).*

Table 8:

*S-fibrinogen intervals, treatment group.*

*Median (IQR), Number (%),* Significant results. Low S-fibrinogen (Fib < 2 g/l) normal S-fibrinogen (Fib 2-3 g/l) and high S-fibrinogen (Fib > 3 g/l). Matching criteria age-gender-ISS at the top. Injury Severity Score (ISS), Activated Partial Thromboplastin Time (APTT), International Normalized Ratio (INR), Platelet count, Glasgow Coma Scale (GCS), Chronic Obstructive Pulmonary Disease (COPD). Continuous variables were analysed with the Mann-Whitney test and categorical variables with Fisher’s exact test.*

Table 9:

*S-fibrinogen, intervals, controls.*

**

*Median (IQR), Number (%),* Significant results. Low S-fibrinogen (Fib < 2 g/l) normal S-fibrinogen (Fib 2-3 g/l) and high S-fibrinogen (Fib > 3 g/l). Matching criteria age-gender-ISS at the top. Injury Severity Score (ISS), Activated Partial Thromboplastin Time (APTT), International Normalized Ratio (INR), Platelet count, Glasgow Coma Scale (GCS), Chronic Obstructive Pulmonary Disease (COPD). Continuous variables were analysed with the Mann-Whitney test and categorical variables with Fisher’s exact test.*

Table 10:

*Outcome, subanalysis coagulopathy.*

**

*Weighted mean, Number (%),* Significant results. Treatment group (Fib+) and controls (Fib-). Criteria of coagulopathy on arrival defined for this study: INR > 1.1 and/or APTT > 40 sec and/or S-fibrinogen < 2 g/l. Red Blood Cells (RBC). Adjusted confounders: age, sex, Injury Severity Score (ISS), S-fibrinogen, Activated Partial Thromboplastin Time (APTT), International Normalized Ratio (INR), Platelet count, hemoglobin, creatinine, , Glasgow Coma Scale (GCS) and penetrating trauma. Adjusted comorbidities: diabetes, hypertension, cardiovascular disease, thromboembolism and Chronic Obstructive Pulmonary Disease (COPD). Continuous variables were analysed with the Mann-Whitney test and categorical variables with Fisher’s exact test.*

Table 11:

*AUC-values for different predictors of mortality (univariate analysis).*

|  | 24 hours-mortality | 30 days-mortality | 1 year-mortality |
| --- | --- | --- | --- |
| GCS | 0.58 (0.41 ; 0.75) | 0.73 (0.63 ; 0.83) 1 | 0.67 (0.57 ; 0.77) 2 |
| APTT | 0.52 (0.24 ; 0.79) | 0.73 (0.64 ; 0.82) 2 | 0.71 (0.62 ; 0.79) 1 |
| S-fibrinogen | 0.64 (0.37 ; 0.90) | 0.64 (0.52 ; 0.76) 3 | 0.59 (0.49 ; 0.69) 3 |
| INR | 0.72 (0.52 ; 0.92) | 0.56 (0.45 ; 0.68) | 0.55 (0.45 ; 0.65) |
| Hemoglobin | 0.65 (0.38 ; 0.91) | 0.53 (0.42 ; 0.65) | 0.55 (0.45 ; 0.66) |
| Platelet count | 0.53 (0.28 ; 0.77) | 0.57 (0.46 ; 0.68) | 0.55 (0.44 ; 0.65) |
| ISS | 0.56 (0.33 ; 0.80) | 0.60 (0.51 ; 0.69) | 0.52 (0.43 ; 0.61) |
| Creatinine | 0.79 (0.63 ; 0.94) 1 | 0.56 (0.45 ; 0.66) | 0.52 (0.42 ; 0.62) |
| SBP | 0.53 (0.30 ; 0.76) | 0.52 (0.39 ; 0.64) | 0.55 (0.45 ; 0.66) |
| Blunt trauma | 0.76 (0.64 ; 0.89) 2 | 0.53 (0.42 ; 0.63) | 0.52 (0.42 ; 0.62) |
| Penetrating trauma | 0.74 (0.60 ; 0.87) 3 | 0.51 (0.41 ; 0.61) | 0.51 (0.42 ; 0.61) |
| Head trauma | 0.66 (0.49 ; 0.83) | 0.50 (0.40 ; 0.59) | 0.53 (0.44 ; 0.62) |

*(95% CI). Creatinine estimated as difference from reference interval (see Statistics section). Best predictor (1), 2:nd best predictor (2), 3:rd best predictor (3). Glasgow Coma Scale (GCS), Activated Partial Thromboplastin Time (APTT), International Normalized Ratio (INR), Platelet count, Injury Severity Score (ISS), Systolic Blood Pressure (SBP).*

Table 12:

*R2-values for different predictors of transfusion (total units of RBC/plasma/thrombocytes/total transfusions).*

|  | RBC | Plasma | Thrombocytes | Total transfusions |
| --- | --- | --- | --- | --- |
| GCS | < 0.001 | < 0.001 | < 0.001 | < 0.001 |
| APTT | < 0.001 | 0.002 | 0.002 | < 0.001 |
| S-fibrinogen | 0.06 | 0.07 (3) | 0.05 (3) | 0.07 (3) |
| INR | 0.09 (2) | 0.02 | 0.01 | 0.04 |
| Hemoglobin | 0.11 (1) | 0.11 (1) | 0.12(1) | 0.12 (1) |
| Platelet count | 0.02 | 0.01 | 0.12 (2) | 0.02 |
| ISS | 0.05 | 0.06 | 0.004 | 0.05 |
| Creatinine | 0.03 | 0.05 | 0.002 | 0.04 |
| SBP | 0.08 (3) | 0.09 (2) | 0.04 | 0.09 (2) |
| Blunt trauma | 0.02 | 0.03 | 0.03 | 0.03 |
| Penetrating trauma | 0.02 | 0.03 | 0.02 | 0.03 |
| Head trauma | 0.001 | < 0.001 | 0.03 | < 0.001 |

*Red Blood Cells (RBC). Best predictor (1), 2:nd best predictor (2), 3:rd best predictor (3). Glasgow Coma Scale (GCS), Activated Partial Thromboplastin Time (APTT), International Normalized Ratio (INR), Platelet count, Injury Severity Score (ISS), Systolic Blood Pressure (SBP).*

Table 13:

*AUC-values for different predictors of mortality (univariate analysis), coagulopathic patients.*

|  | 24 hours-mortality | 30 days-mortality | 1 year-mortality |
| --- | --- | --- | --- |
| GCS | 0.61 (0.42 ; 0.80) | 0.73 (0.63 ; 0.84) 1 | 0.64 (0.53 ; 0.75) 3 |
| APTT | 0.31 (0.02 ; 0.60) 3 | 0.70 (0.60 ; 0.80) 2 | 0.69 (0.59 ; 0.79) 1 |
| S-fibrinogen | 0.42 (0.10 ; 0.74) | 0.68 (0.55 ; 0.80) 3 | 0.59 (0.47 ; 0.71) |
| INR | 0.53 (0.22 ; 0.83) | 0.44 (0.31 ; 0.57) | 0.42 (0.31 ; 0.54) |
| Hemoglobin | 0.65 (0.43 ; 0.87) | 0.59 (0.48 ; 0.69) | 0.50 (0.40 ; 0.60) |
| Platelet count | 0.28 (0.02 ; 0.54) 2 | 0.51 (0.40 ; 0.62) | 0.57 (0.46 ; 0.68) |
| ISS | 0.46 (0.27 ; 0.65) | 0.62 (0.52 ; 0.72) | 0.60 (0.50 ; 0.71) |
| Creatinine | 0.16 (0.04 ; 0.27) 1 | 0.39 (0.30 ; 0.49) | 0.32 (0.23 ; 0.42) 2 |
| SBP | 0.50 (0.27 ; 0.74) | 0.60 (0.47 ; 0.73) | 0.60 (0.47 ; 0.73) |
| Blunt trauma | 0.63 (0.41 ; 0.86) | 0.51 (0.39 ; 0.62) | 0.52 (0.41 ; 0.62) |
| Penetrating trauma | 0.35 (0.12 ; 0.58) | 0.50 (0.38 ; 0.62) | 0.50 (0.38 ; 0.61) |
| Head trauma | 0.47 (0.27 ; 0.68) | 0.36 (0.25 ; 0.46) | 0.37 (0.27 ; 0.47) |

*(95% CI). Criteria of coagulopathy on arrival defined for this study: INR > 1.1 and/or APTT > 40 sec and/or S-fibrinogen < 2 g/l. Creatinine estimated as difference from reference interval (see Statistics). Best predictor (1), 2:nd best predictor (2), 3:rd best predictor (3). Glasgow Coma Scale (GCS), Activated Partial Thromboplastin Time (APTT), International Normalized Ratio (INR), Platelet count, Injury Severity Score (ISS), Systolic Blood Pressure (SBP).*

Figure 4:


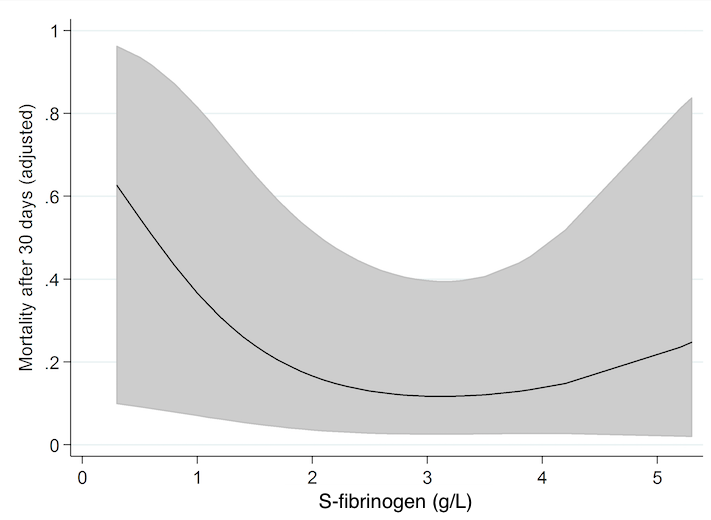


*30 days-mortality vs. S-fibrinogen (g/l), all patients. Data adjusted for confounders (age, Injury Severity Score (ISS), Activated Partial Thromboplastin Time (APTT), International Normalized Ratio (INR), hemoglobin, creatinine, Glasgow Coma Scale (GCS).*
